# Supplementary material for: Chitosan as an Alternative to Oil-Based Materials for the Fabrication of Lab-on-a-Chip
Source: Micromachines (Basel). 2024 Mar 12;15(3):379. doi: 10.3390/mi15030379 (PMC10971945; doi:10.3390/mi15030379)
Supplement: Supplementary file 1 [file micromachines-15-00379-s001.zip › micromachines-2891335-supplementary.pdf]

Supplementary Materials for

Chitosan as an alternative to oil-based materials for the fabrication of lab-on-a-chip

Morgane Zimmer [1,*], Stéphane Trombotto [2], Emmanuelle Laurenceau [1] and Anne-Laure Deman [1]

[1] Ecole Centrale de Lyon, INSA Lyon, CNRS, Université Claude Bernard Lyon 1, CPE Lyon, INL, UMR5270, 69130 Ecully, France; emmanuelle.laurenceau@ec-lyon.fr (E.L.); anne-laure.deman-him@univ-lyon1.fr (A.-L.D.)

[2] Ingénierie des Matériaux Polymères (UMR5223), Université Claude Bernard Lyon 1, CNRS, INSA Lyon, Université Jean Monnet, 69622 Villeurbanne, France; stephane.trombotto@univ-lyon1.fr

* Correspondence: morgane.zimmer@ec-lyon.fr

Characterization Methods of Starting Chitosan (244LG, Batch Number 20140503, Mahtani Chitosan Pvt. Ltd., Veraval, India)

1. Proton Nuclear Magnetic Resonance Spectroscopy (^{1}H NMR)

The DA of starting chitosan was determined by ^{1}H NMR according to the method described by Hirai et al. [75]. The ^{1}H NMR spectrum was recorded on an AV300 Bruker (300 MHz) spectrometer (Bruker, Billerica, MA, USA) at ambient temperature. Chitosan powder was dissolved at 10 mg/mL in D_2O with 5 μL HCl (12 N) and transferred to 5 mm NMR tubes. Trimethylsilyl-3-propionic-2,2,3,3-D4 acid sodium salt (TMPSA, Sigma-Aldrich, Saint-Quentin Fallavier, France) was used as an internal reference. The Bruker Topspin software (Bruker, Billerica, MA, USA, version 3.6) was used for the analysis of the spectrum.

2. Size-Exclusion Chromatography (SEC)

The average molar masses (Mw and Mn) and the dispersity Đ of starting chitosan were determined by size exclusion chromatography (SEC). Chitosan powder was first dissolved in a 0.2 M acetic acid/0.15 M ammonium acetate buffer (pH 4.5) at a concentration from 1.0 mg/mL for a minimum of 18 h at ambient temperature. The solution was then filtered using 0.45 μm pore size CME membranes (Millipore, Burlington, MA, USA). The macromolecule separation was performed on two serially connected columns (TSK G2500PW and TSK G6000PW, Tosoh Bioscience, Tokyo, Japan). A differential refractometer (Optilab T-rex, Wyatt Technology, Santa Barbara, USA) coupled online with a MALLS detector (Dawn Heleos II, Wyatt Technology, Santa Barbara, USA) was used for the detection. A degassed 0.2 M acetic acid/0.15 M ammonium acetate buffer (pH 4.5) was used as an eluent after filtration on a 0.10 μm pore size membrane (Millipore, Burlington, MA, USA). The flow rate was maintained at 0.5 mL/min, and the amount of sample injected was 100 μL. The refractive index increment (dn/dc) was adjusted to 0.198 for a degree of acetylation of 0.8% according to the results of Schatz et al. [76]. The ASTRA software (Wyatt Technology, Santa Barbara, USA, version 6.1) was used for the analysis of chromatogram.

3. Thermogravimetric Analysis (TGA)

Water and ash contents of starting chitosan were determined by TGA using an SDT-Q600 analyzer (TA Instruments, New Castle, DE, USA). Thermogravimetric analysis was performed with 15-20 mg of sample under a flow of air (100 mL/min), and by using a temperature ramp of 2 °C/min from ambient temperature to 200 °C, then a temperature ramp of 20 °C/min up to 900 °C and finally an isotherm of

30 min at 900 °C. The TA Universal Analysis 2000 software (TA Instruments, New Castle, DE, USA, version 4). was used for the analysis of thermogram.

References

75. Hirai, A.; Odani, H.; Nakajima, A. Determination of Degree of Deacetylation of Chitosan by 1H NMR Spectroscopy. Polym. Bull. 1991, 26, 87–94. https://doi.org/10.1007/BF00299352
76. Schatz, C.; Viton, C.; Delair, T.; Pichot, C.; Domard, A. Typical Physicochemical Behaviors of Chitosan in Aqueous Solution. Biomacromolecules 2003, 4 (3), 641–648. https://doi.org/10.1021/bm025724c

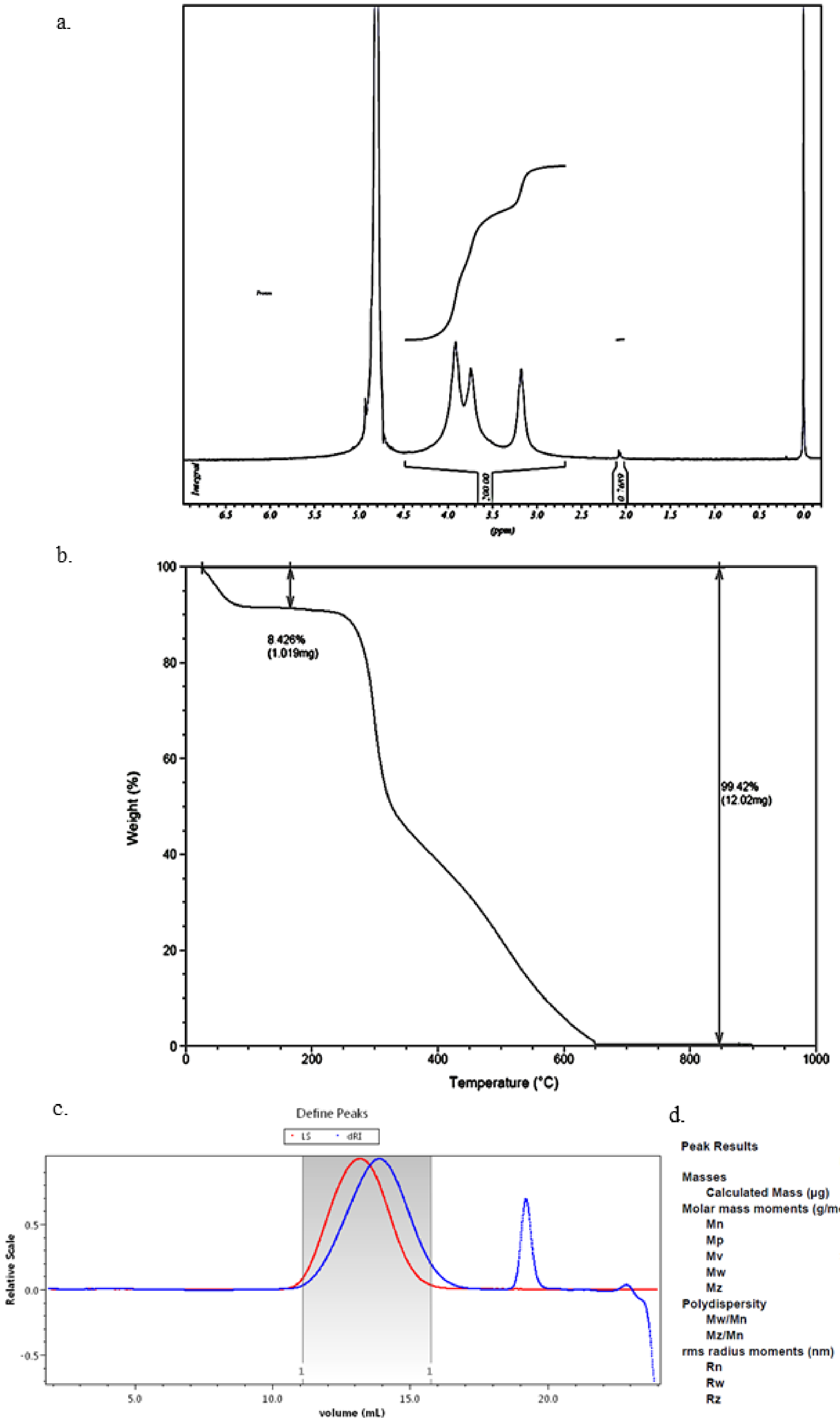

d.

Peak Results

	Peak 1
**Masses**	
Calculated Mass (µg)	91.20
**Molar mass moments (g/mol)**	
Mn	1.043×10^5 (±1.246%)
Mp	1.365×10^5 (±0.264%)
Mv	n/a
Mw	1.925×10^5 (±0.761%)
Mz	3.221×10^5 (±2.671%)
**Polydispersity**	
Mw/Mn	1.846 (±1.460%)
Mz/Mn	3.087 (±2.948%)
**rms radius moments (nm)**	
Rn	37.1 (±4.1%)
Rw	51.4 (±1.4%)
Rz	67.1 (±1.1%)

Figure S1. Characterization of starting chitosan (244LG, batch number 20140503, Mahtani Chitosan Pvt. Ltd., Veraval, India) by: (a) ^{1}H NMR spectroscopy for the determination of DA, (b) Thermogravimetric analysis for the determination of water and ash contents and (c and d) Size-exclusion chromatography of the determination of average molar masses (Mw and Mn) and dispersity (Mw/Mn).

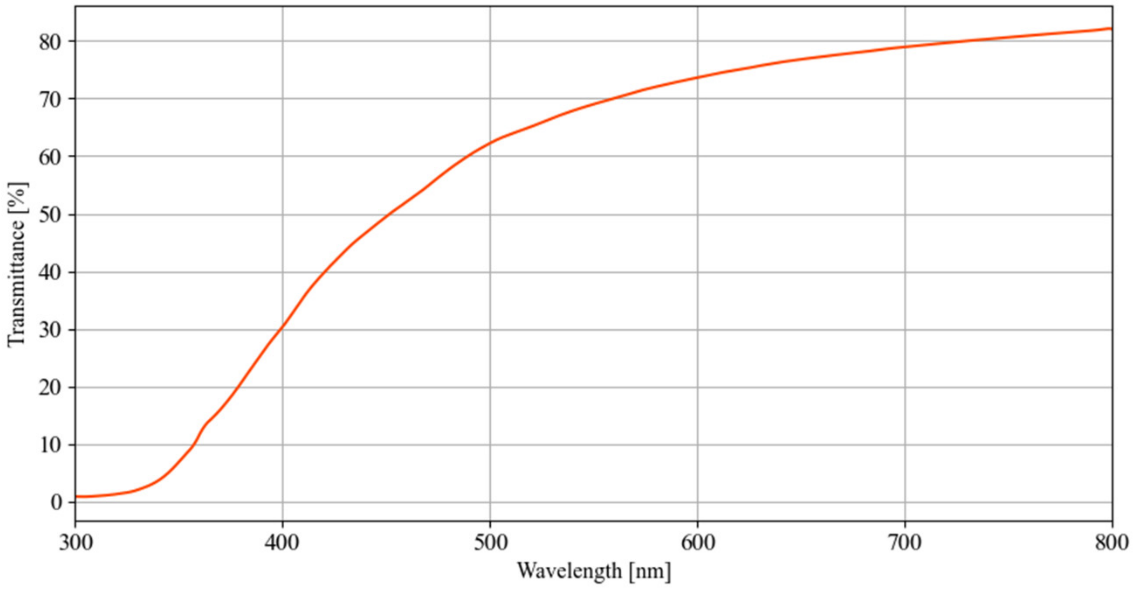

Figure S2. Transmittance spectrum of 0.3 mm thick neutralized chitosan film in the range of 300 to 800 nm.

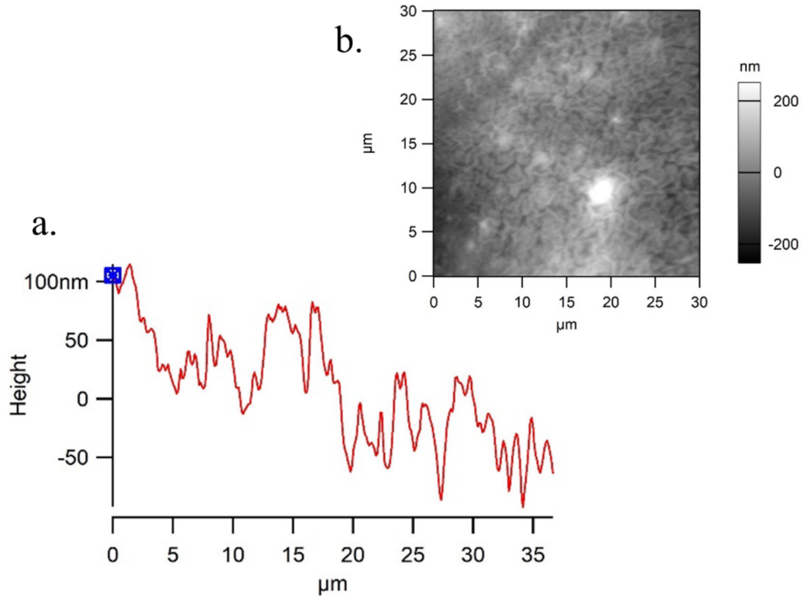

Figure S3. AFM measurements on neutralized chitosan film: Line measurement (a) of the height extracted from the height map (b)

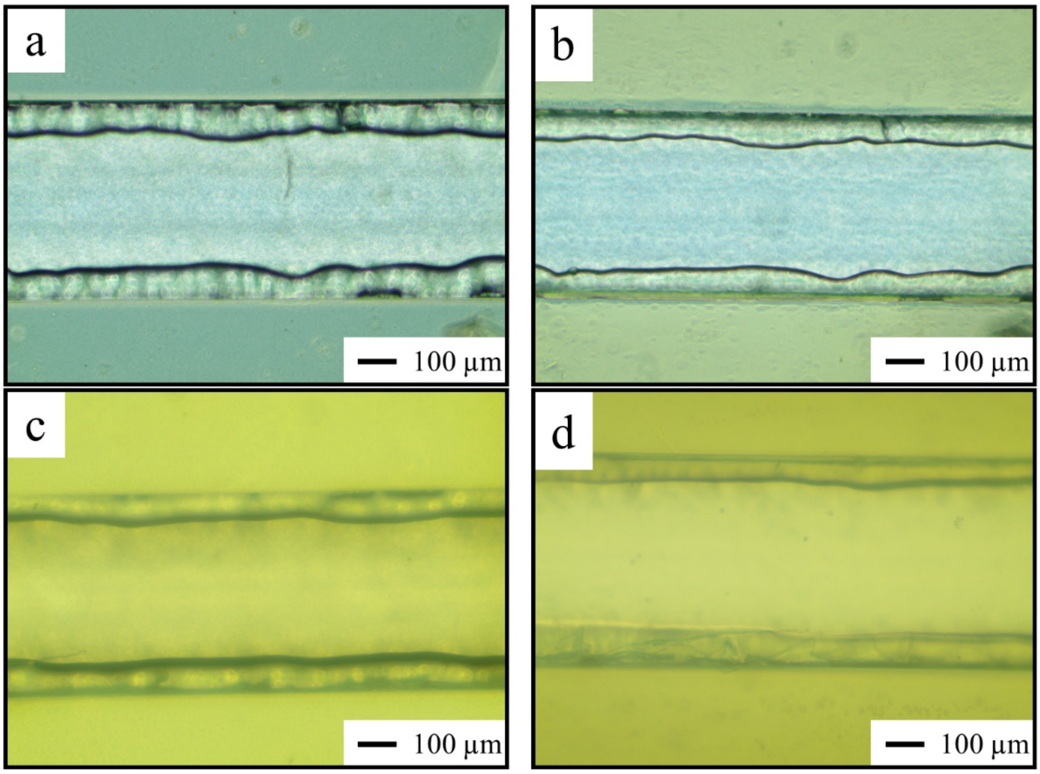

Figure S4: Microscopic images of a chitosan/glass device with a 500 µm wide and 150 µm high channel before the injection (a) and after 24 h of injection of deionized water at 100 µL.h-1 (b); Images of an all-chitosan device before (c) and after 24 h of injection (d).
